# Supplementary material for: Identification and characterization of small molecule inhibitors of the LINE-1 retrotransposon endonuclease
Source: Nat Commun. 2024 May 8;15:3883. doi: 10.1038/s41467-024-48066-x (PMC11078990; doi:10.1038/s41467-024-48066-x)
Supplement: Supplementary file 1 — Supplementary Information [file 41467_2024_48066_MOESM1_ESM.pdf]

Identification and characterization of small molecule inhibitors of the LINE-1 retrotransposon endonuclease

Alexandra M. D'Ordine, Gerwald Jogl, John M. Sedivy

Supplementary Information

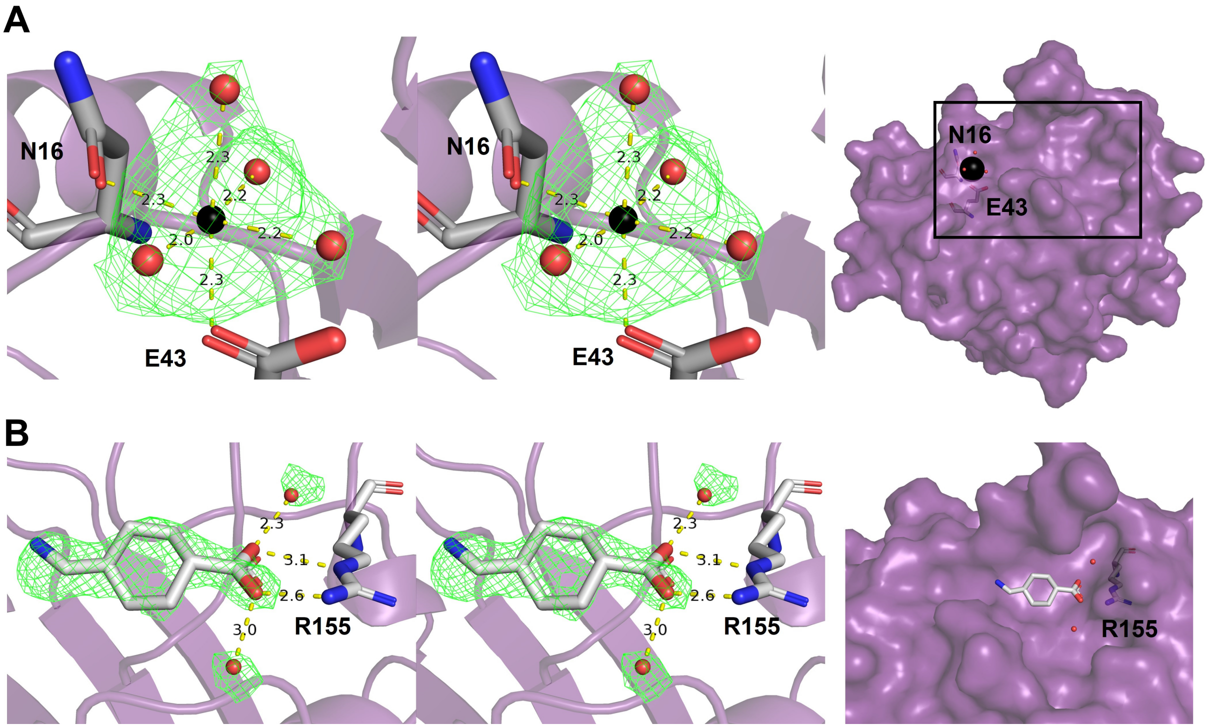

Supplementary Figure 1: EN crystal structures solved and used for docking. A) EN structure with Mn^{2+} ion bound (PDB ID: 8SP5). Left: stereo view of structure with Fo-Fc electron density omit difference map (green, contoured at 3σ) surrounding Mn^{2+} (black) and waters (red). Mn^{2+} coordination by waters and active site residues (grey) is shown with distances (Å) indicated. Right: surface view with DNA binding site and approximate area used for docking indicated with black box, with Mn^{2+}-interacting residues and waters shown. **B)** EN structure with fragment tranexamic acid bound (PDB ID: 8SP7). Left: stereo view of structure with Fo-Fc electron density omit difference map (green, contoured at 3σ) surrounding tranexamic acid (grey) and waters (red). Coordination by waters and active site residue (grey) is shown with distances (Å) indicated. Right: surface view of DNA binding site containing tranexamic acid, with interacting residue and waters shown.

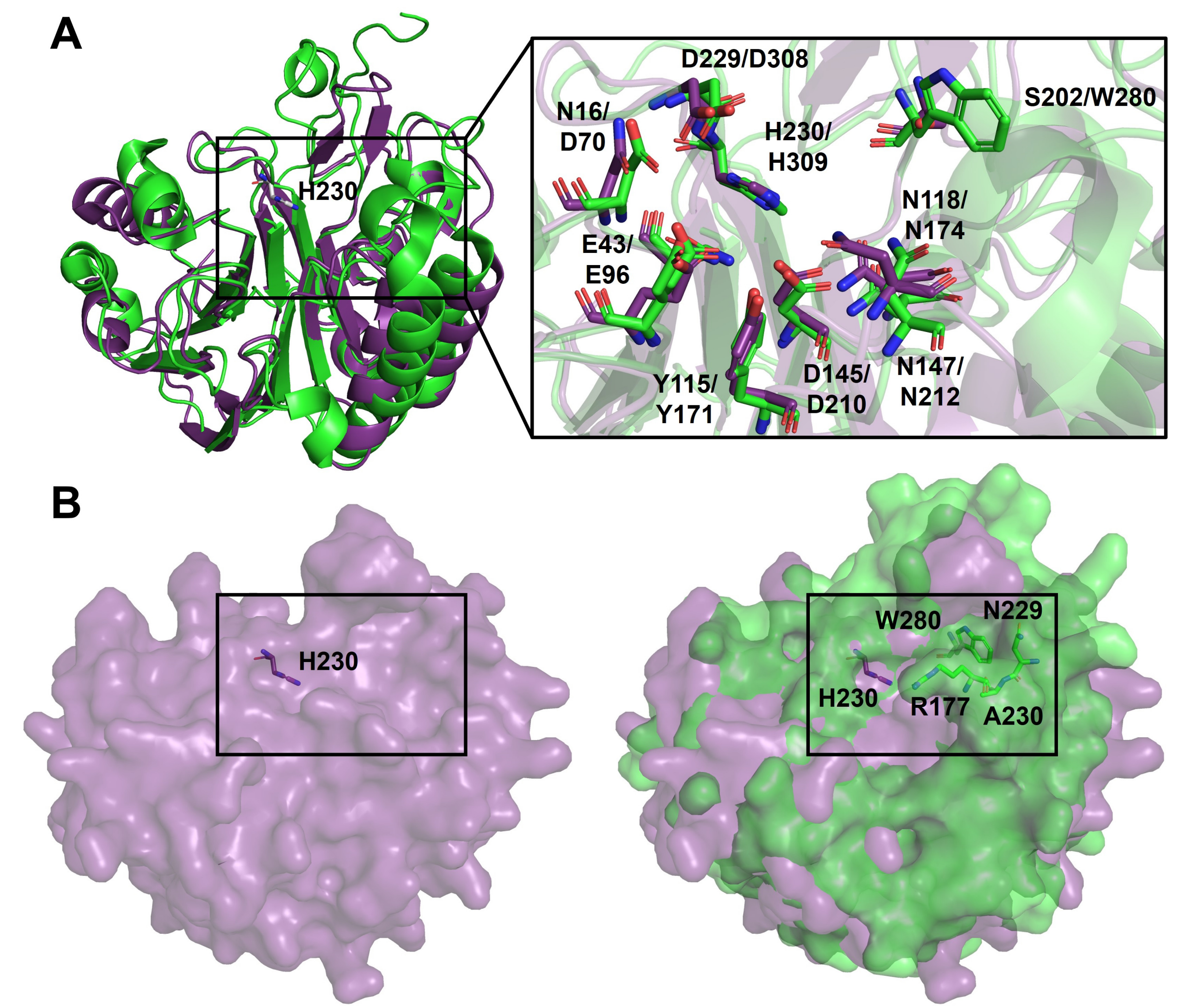

Supplementary Figure 2: EN and APE1 structural comparison. EN (PDB ID: 1VYB, purple) and APE1 (PDB ID: 4QHE, green) structures were superimposed in PyMOL. **A)** Comparison of EN and APE1 active site residues. Cartoon view of full structures at left with EN catalytic residue H230 shown. Inset view at right showing approximate area used for docking and indicating selected residues involved in catalysis, metal ion coordination, or DNA binding. Residue number in the EN listed first. **B)** Differences between the APE1 and EN DNA binding surfaces. Left: approximate area of DNA binding site used for docking is marked by a black box and residue H230 is shown for reference. Right: superimposition of EN and APE1 surfaces with key differing residues present in APE1 only indicated, as well as H230 in EN for reference.

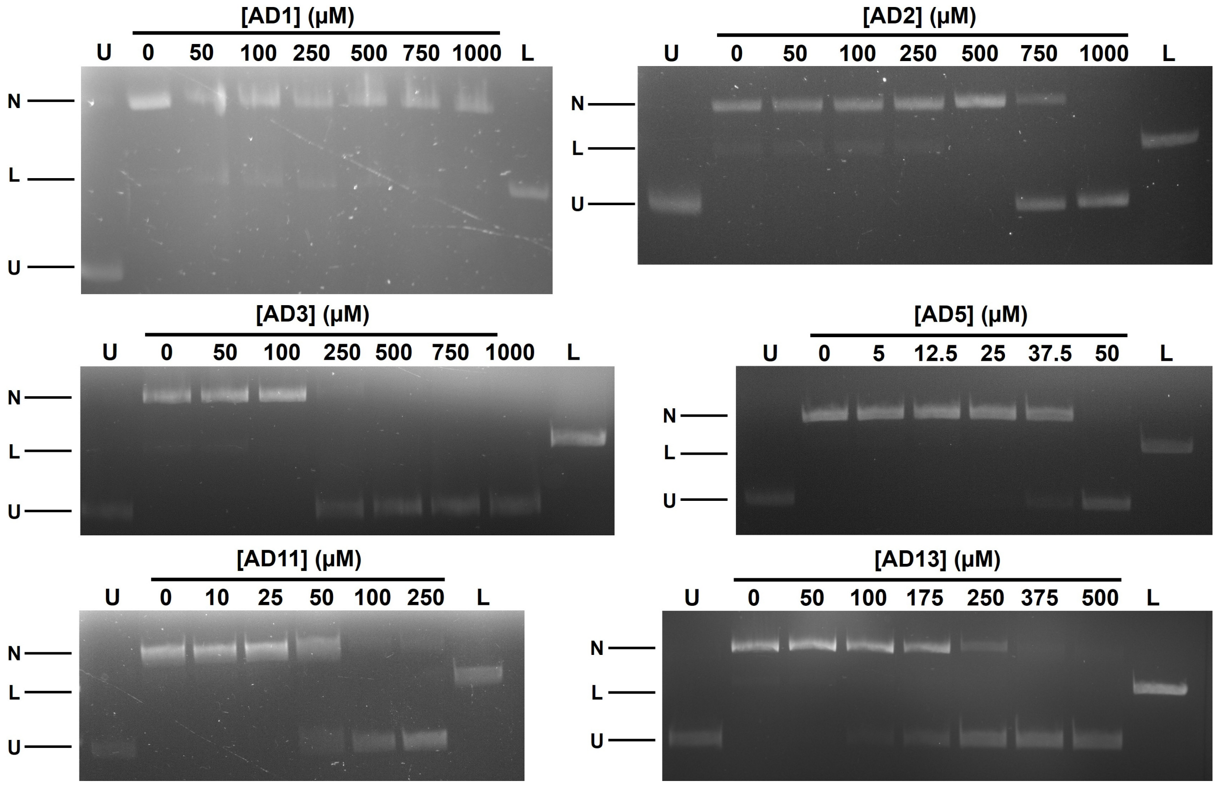

Supplementary Figure 3: Plasmid nicking assay results for selected compounds. Initial candidate compounds were screened using this previously developed EN activity assay. Activity is indicated by slower migration of plasmid on the agarose gel due to nicking and subsequent loss of supercoiling. U, uncut, supercoiled plasmid; L, linearized plasmid; N, nicked plasmid. Similar images were obtained from at least 2 independent experiments. Uncropped gel images are provided as a Source Data file.

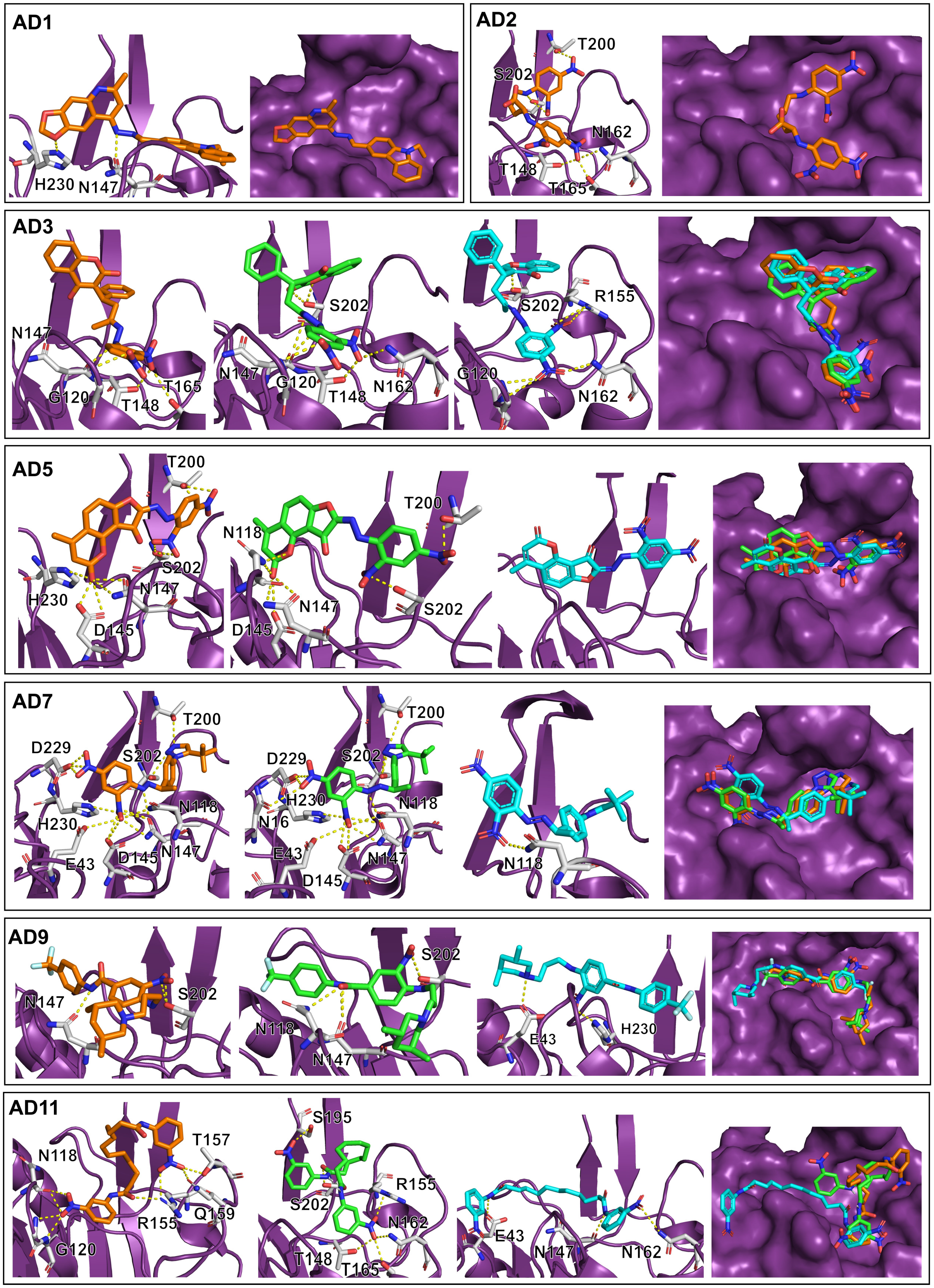

Supplementary Figure 4: EN inhibitor docking results (legend below).

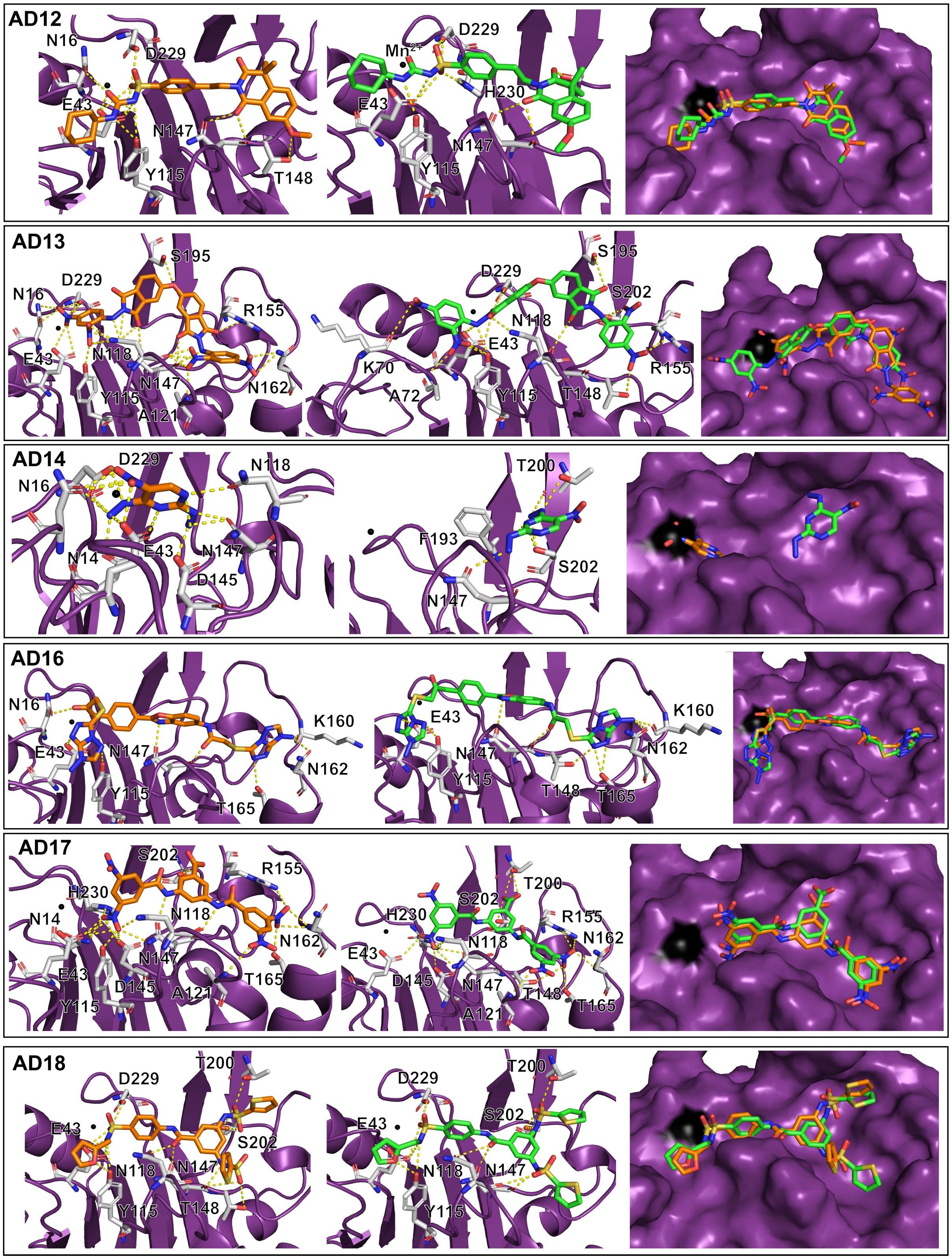

Supplementary Figure 4: EN inhibitor docking results (legend below).

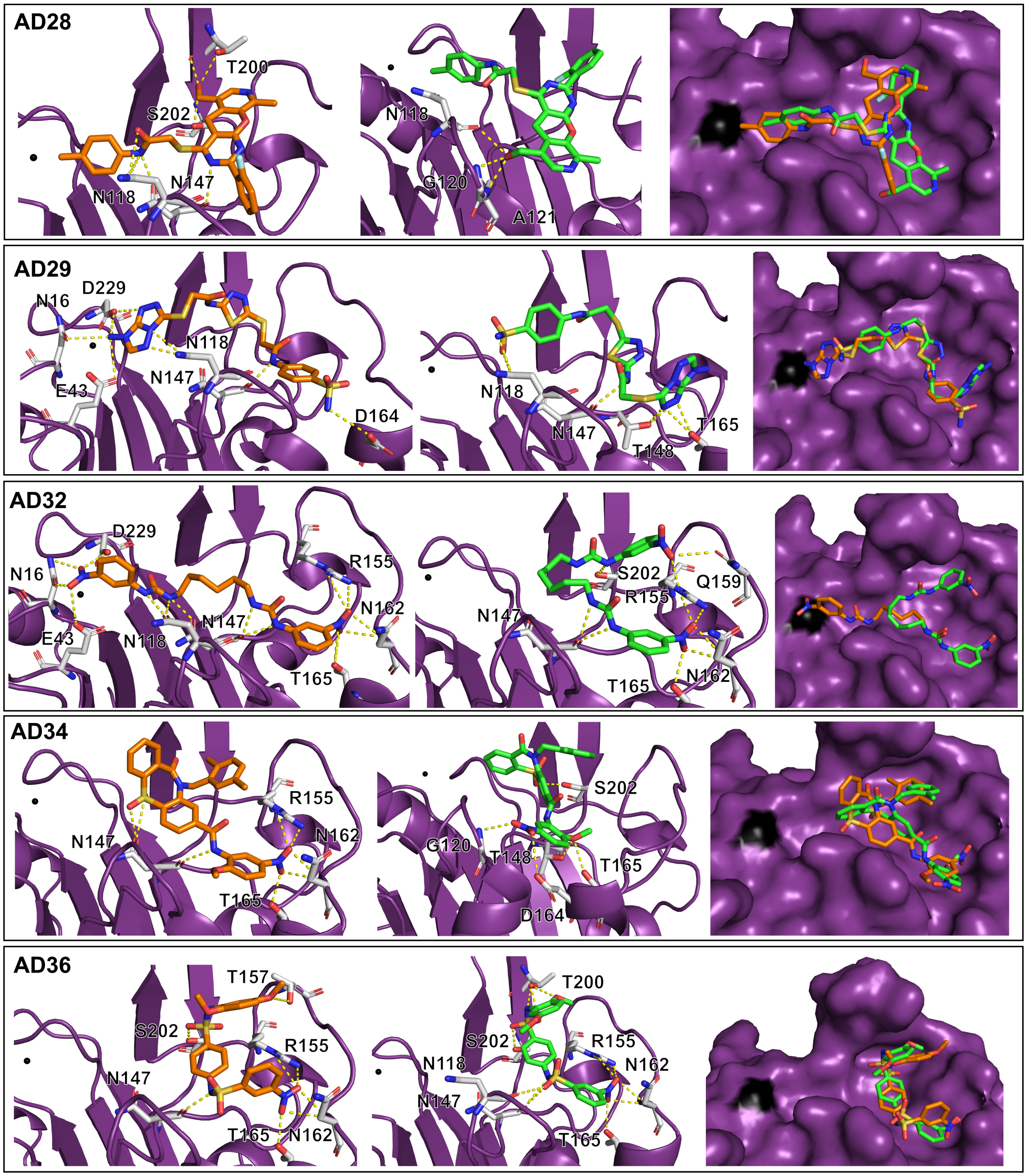

Supplementary Figure 4: EN inhibitor docking results (legend below).

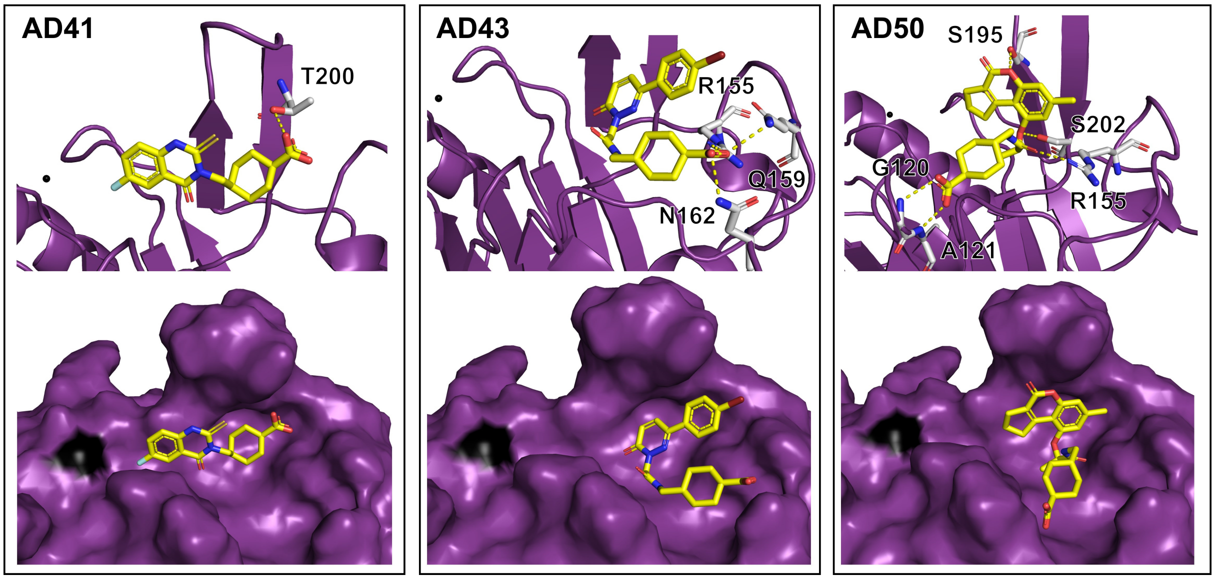

Supplementary Figure 4: EN inhibitor docking results. Docking results for all EN inhibitors described in this work, as well as AD1. Polar interactions between inhibitors and residues as identified by PyMOL are indicated with dashed yellow lines, and the Mn^{2+} ion is shown in black where applicable. Compound structures are colored by program and overlaid in surface view to allow for comparison of results among programs where applicable: LeDock, orange; AutoDock Vina, green; DOCK 6.9, light blue; FitDock, yellow.

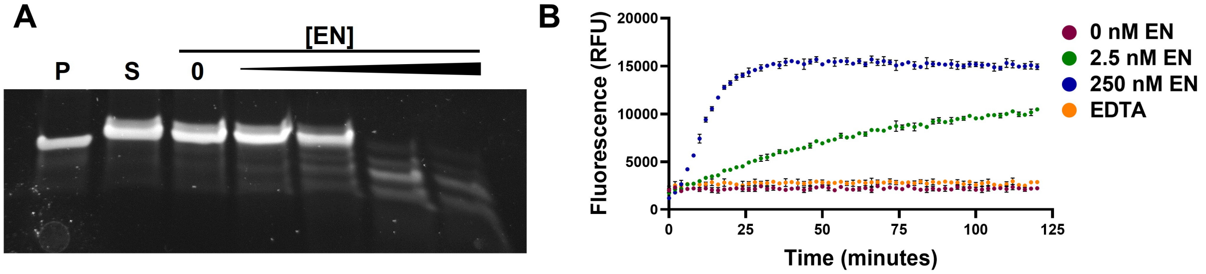

Supplementary Figure 5: Fluorescent oligonucleotide EN activity assay development. A) Denaturing PAGE gel showing nicking of 43-nucleotide substrate hairpin with increasing concentration of EN. P, 35-nucleotide product; S, 43-nucleotide unannealed substrate oligonucleotide. Similar images were obtained from two independent experiments. **B)** Fluorescence over time resulting from EN activity measured by a plate reader. 25 nM substrate was incubated with indicated concentrations of EN. EDTA control condition contained 50 mM EDTA and 2.5 nM EN. Data are mean ± s.d. from triplicate wells (n=3) for each condition. Uncropped gel image and source data are provided as a Source Data file.

A

Name	APE1 activity (%)	Name	APE1 activity (%)	Name	APE1 activity (%)
AD1	0.5 ± 0.3	AD12	109.1 ± 5.2	AD29	81.2 ± 2.4
AD2	96.2 ± 3.4	AD13	84.4 ± 15.2	AD32	107.9 ± 2.8
AD3	103.5 ± 10.4	AD14	78.1 ± 6.6	AD34	113.8 ± 3.1
AD5	109.3 ± 1.9	AD16	95.5 ± 4.2	AD36	118.2 ± 11.9
AD7	118.8 ± 4.7	AD17	51.1 ± 8.7	AD41	111.5 ± 7.9
AD9	115.9 ± 3.1	AD18	103.1 ± 9.2	AD43	115.9 ± 7.4
AD11	79.4 ± 8.9	AD28	118.3 ± 1.2	AD50	115.7 ± 2.8

B

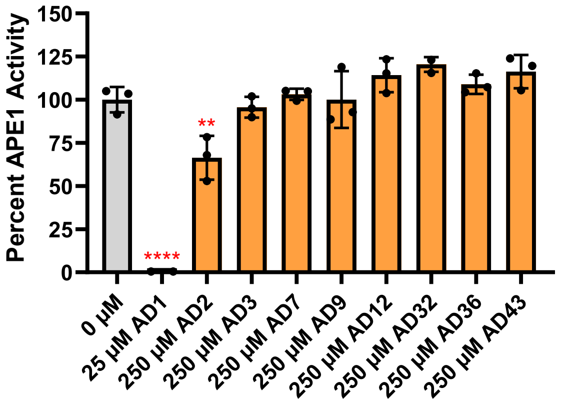

Supplementary Figure 6: APE1 assay testing EN inhibitors. **A)** Table of APE1 percent activities relative to 0 µM inhibitor control for inhibitors at 25 µM. Percent activity values were calculated from 3 independent experiments with 3 replicates each and are mean ± s.d. **B)** Percent activity of APE1 incubated with indicated inhibitors. Statistical significance of the mean relative to 0 µM inhibitor was determined by one-way ANOVA followed by Dunnett's multiple comparisons test using GraphPad Prism: *p<0.05, **p<0.01, ***p<0.001, ****p<0.0001. Data are mean ± s.d (n=3 samples). Source data and exact p-values are provided as a Source Data file.

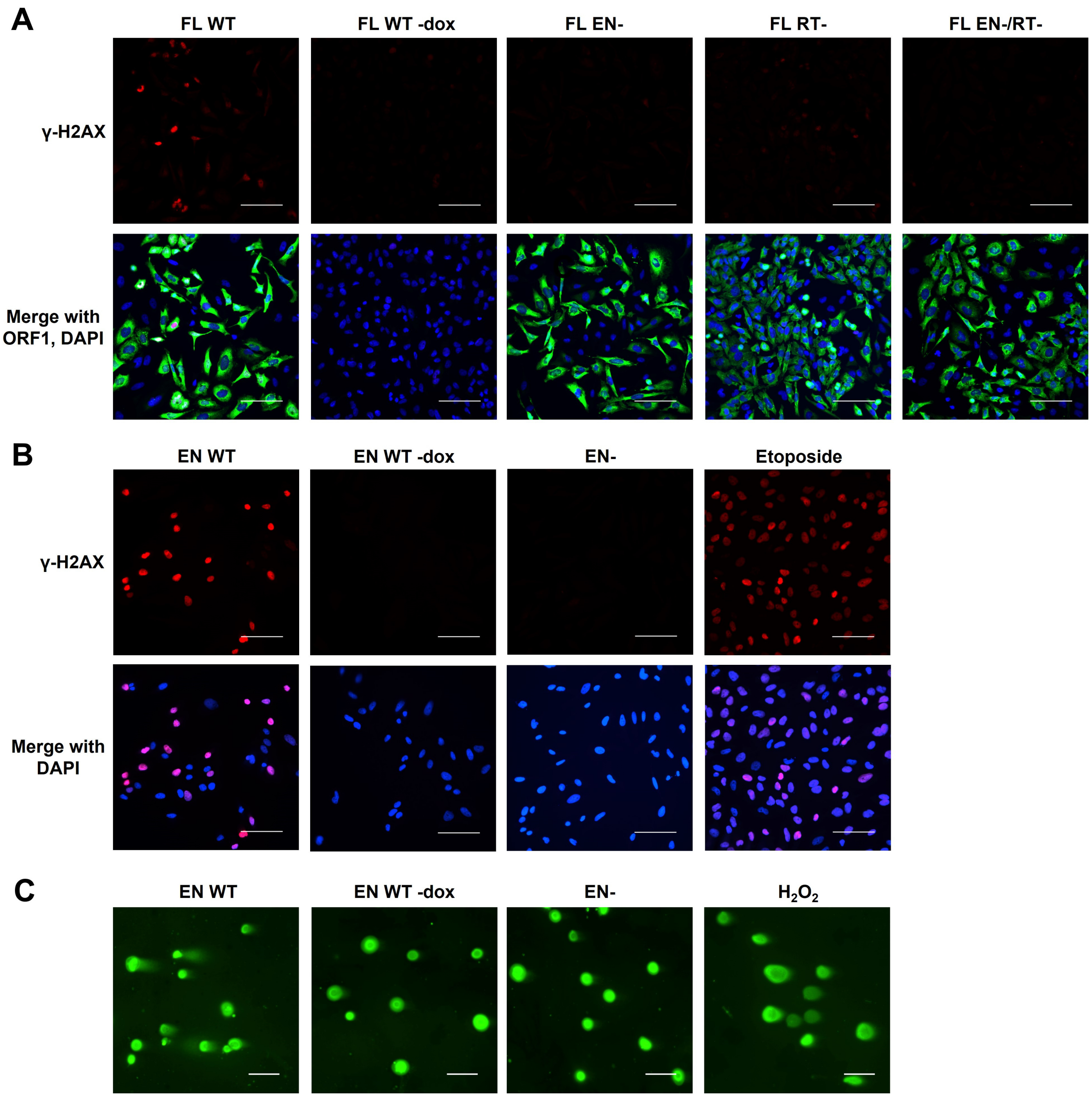

Supplementary Figure 7: Fluorescence microscopy images from DNA damage assays. **A)** Representative immunofluorescence images of HeLa cells with full-length (FL) L1 constructs induced or not induced with doxycycline (dox), stained for γ-H2AX (red) and ORF1 (green) as indicated. **B)** Representative immunofluorescence images of HeLa cells with EN constructs induced, not induced, or treated with etoposide, stained for γ-H2AX (red) as indicated. **C)** Representative fluorescence microscopy images of neutral comet assay results for HeLa cells expressing EN domain constructs, not induced, or treated with hydrogen peroxide as indicated, all stained with SYBR Gold. Similar images were obtained from at least two independent experiments. Scale bar=100 μm.

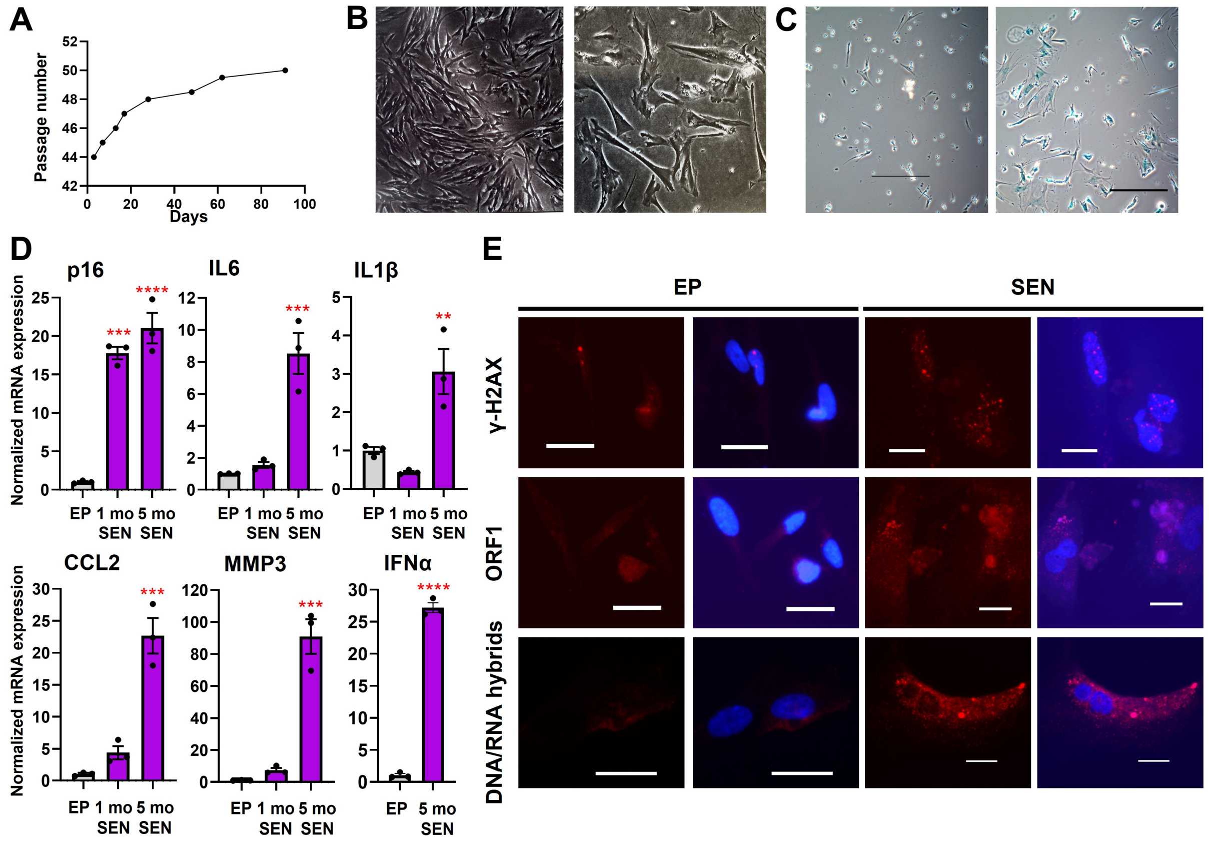

Supplementary Figure 8: Replicative senescence cell culture model of human diploid fibroblasts (LF1). A) Representative passaging history showing progressive slowing of cell division as cells enter senescence (SEN). **B)** Images of LF1 cells in culture, with cell enlargement indicating senescence. Left: early passage (EP) proliferating control. Right: SEN entry (0 months SEN). Similar images were obtained from three independent experiments**. C)** Senescence-associated β-galactosidase assay, with blue staining indicating senescence. Similar images were obtained from two independent experiments**.** Left: EP. Right: 1 month SEN. Scale bar=500 µm. **D)** Representative RT-qPCR results of p16, SASP markers IL6, IL1β, CCL2, and MMP3, and IFN-I marker IFNα throughout senescence normalized to EP. Statistical significance of the mean relative to EP was determined by one-way ANOVA followed by Dunnett's multiple comparisons test or unpaired two-tailed *t*-test (IFNα only) using GraphPad Prism: *p<0.05, **p<0.01, ***p<0.001, ****p<0.0001. Data are mean ± s.e.m. **E)** Immunofluorescence images of EP and SEN cells stained for indicated markers (γ-H2AX and ORF1, 3 months SEN; DNA/RNA hybrids, 4 months SEN). Scale bar=25 µm. Similar images were obtained from at least two independent experiments**.** Source data and exact p-values are provided as a Source Data file.

Supplementary Table 1: Data collection and refinement statistics for EN crystal structures[a]

	EN complex with Mn^{2+}	EN complex with tranexamic acid
**Data collection**		
Space group	P 2_1 2_1 2	C 2 2 2_1
Cell dimensions		
a, *b*, *c* (Å)	93.10, 122.38, 43.41	54.22, 64.67, 132.36
α, β, γ (°)	90.00, 90.00, 90.00	90.00, 90.00, 90.00
Resolution (Å)	46.55-2.23 (2.30-2.23)	29.05-1.83 (1.87-1.83)
R_{merge}	0.195 (1.251)	0.088 (1.138)
$CC_{1/2}$	0.996 (0.853)	0.998 (0.897)
$I/\sigma I$	9.2 (2.2)	13.9 (2.1)
Completeness (%)	100 (100)	100 (100)
Multiplicity	13.5 (12.4)	13.6 (14.2)
**Refinement**		
Resolution (Å)	2.23	1.83
No. reflections	25007	20883
R_{work} / R_{free}	0.203/0.250	0.220/0.256
No. atoms		
Protein	3758	1819
Ligand/ion	27	30
Water	236	61
B-factors		
Protein	37.37	50.65
Ligand/ion	43.55	60.08
Water	38.09	51.24
R.m.s. deviations		
Bond lengths (Å)	0.007	0.006
Bond angles (°)	0.89	0.87
Ramachandran favored (%)	98.28	96.12
Ramachandran allowed (%)	1.72	3.45
Ramachandran outliers (%)	0.00	0.43
Rotamer outliers (%)	1.64	0.51

[a] One crystal was used for each structure.

Supplementary Table 2: EN inhibitor and oligonucleotide binding by spectral shift. EN inhibitors were incubated with the substrate oligonucleotide used in the EN activity assay and labeled with Cy5. The change in spectral shift ratio between the oligonucleotide with and without each inhibitor (response amplitude), as well as the signal to noise ratio of this response, was measured and used by the MO.Control software (NanoTemper) to determine the presence or absence of binding. Inhibitors were tested at the highest soluble concentration in spectral shift assay buffer.

Compound	Response Amplitude	Signal to Noise Ratio	Binding Detected? (Y/N)
SYBR Gold	0.101	79.39	Y
AD2	0.003	1.811	N
AD3	0.004	0.373	N
AD5	0.009	3.487	N
AD7	0.009	2.553	N
AD9	0.003	0.398	N
AD11	0.012	1.936	N
AD12	0.014	3.485	N
AD13	0.005	1.436	N
AD14	0.012	1.282	N
AD16	0.019	8.275	Y
AD17	0.004	2.000	N
AD18	0	0.170	N
AD28	0.004	2.501	N
AD29	0.006	2.466	N
AD32	0.001	0.839	N
AD34	0.001	0.667	N
AD36	0.014	4.209	N
AD41	0.001	0.865	N
AD43	0.017	2.642	N
AD50	0.003	2.854	N

Supplementary Table 3: Summary of DNA damage assay replicates. Summary of independent experiments of γ-H2AX and comet assays (Fig. 5) reporting means of γ-H2AX intensities or comet tail lengths of individual nuclei (n) normalized to WT. Average intensity or tail length and associated standard deviation across replicates is also shown. Statistical significance of the mean relative to WT was calculated by one-way ANOVA followed by Dunnett's multiple comparisons test using GraphPad Prism. Source data are provided as a Source Data file.

**FL γ-H2AX**	Replicate 1			Replicate 2			Replicate 3			Replicate 4			Summary	
Treatment	mean	p-value	n	mean	p-value	n	mean	p-value	n	mean	p-value	n	average	s.d.
WT	1		117	1		83	1		143	1		120	1	
-dox	0.421	<0.0001	15	0.595	<0.0001	52	0.613	<0.0001	130	0.543	<0.0001	16	0.543	0.087
EN-	0.395	<0.0001	21	0.703	<0.0001	46	0.644	<0.0001	197	0.743	0.0302	15	0.621	0.156
RT-	0.453	<0.0001	80	0.634	<0.0001	280	0.598	<0.0001	325	0.657	<0.0001	67	0.586	0.091
EN-/RT-	0.361	<0.0001	41	0.588	<0.0001	113	0.567	<0.0001	413	0.602	<0.0001	26	0.529	0.113
10 μM 3TC	0.742	<0.0001	168	0.671	<0.0001	71	0.740	<0.0001	173				0.718	0.040
20 μM AD3	0.752	<0.0001	104	0.782	0.0006	38	0.727	<0.0001	336				0.754	0.028
20 μM AD7	0.673	<0.0001	107	0.982	>0.9999	107	0.987	>0.9999	197				0.881	0.180
50 μM AD11				0.738	<0.0001	62	0.875	0.0009	151	0.790	0.0006	56	0.801	0.069
50 μM AD12	0.742	<0.0001	120	0.704	<0.0001	25	0.777	<0.0001	155				0.741	0.037
50 μM AD14	0.740	0.0005	62	0.670	<0.0001	85	0.909	0.0181	215				0.773	0.123
50 μM AD16	0.788	0.0349	41				0.872	0.0002	220	0.967	0.9719	169	0.876	0.090
50 μM AD29				0.655	<0.0001	64	0.742	<0.0001	199	0.722	<0.0001	116	0.707	0.046
5 μM AD32				0.822	0.0008	68	0.788	<0.0001	195	0.922	0.6214	58	0.844	0.070

**EN γ-H2AX**	Replicate 1			Replicate 2			Replicate 3			Replicate 4			Replicate 5			Summary	
Treatment	mean	p-value	n	mean	p-value	n	mean	p-value	n	mean	p-value	n	mean	p-value	n	average	s.d.
WT	1	N/A	257	1	N/A	301	1	N/A	257	1	N/A	214	1	N/A	544	1	
no dox				0.337	<0.0001	200	0.461	<0.0001	619	0.365	<0.0001	346	0.207	<0.0001	804	0.342	0.105
EN-	0.292	0.0096	42	0.350	<0.0001	363	0.083	<0.0001	323	0.345	<0.0001	442	0.193	<0.0001	668	0.253	0.114
20 μM AD3	0.769	0.8621	51	0.730	<0.0001	135	0.624	<0.0001	174							0.708	0.075
20 μM AD7	0.630	0.2813	67	0.830	0.0116	117							0.852	0.0063	322	0.771	0.122
50 μM AD11	0.637	0.359	57	0.615	<0.0001	109	0.633	<0.0001	106							0.628	0.011
100 μM AD12	0.522	0.1429	48	0.883	0.1349	142	0.327	<0.0001	136							0.577	0.282
50 μM AD14	0.779	0.7691	88	0.662	<0.0001	116	0.414	<0.0001	133							0.618	0.187
25 μM AD16	0.656	0.3427	71	0.791	<0.0001	204	0.325	<0.0001	60							0.591	0.240
100 μM AD29	0.409	0.0308	50	0.534	<0.0001	75	0.903	0.7755	126				0.691	<0.0001	242	0.634	0.213
5 μM AD32				0.516	<0.0001	111	0.604	<0.0001	160	0.604	<0.0001	159				0.574	0.051

**EN comet**	Replicate 1			Replicate 2			Replicate 3			Replicate 4			Summary	
Treatment	mean	p-value	n	mean	p-value	n	mean	p-value	n	mean	p-value	n	average	s.d.
WT	1		54	1		117	1		62	1		46	1	
no dox	0.661	0.0484	56	0.612	0.0019	127	0.39	<0.0001	90	0.326	<0.0001	83	0.498	0.163
EN-	0.643	0.0238	67	0.532	<0.0001	166	0.36	<0.0001	82	0.559	0.0003	76	0.524	0.117
20 μM AD3				0.679	0.0328	98	0.57	0.0062	59	0.482	0.0005	34	0.576	0.099
20 μM AD7				0.573	0.0009	107	0.81	0.6224	59	0.310	<0.0001	48	0.566	0.252
50 μM AD11	0.605	0.0364	37	0.832	0.6311	90	0.81	0.6442	52	0.384	<0.0001	40	0.658	0.209
50 μM AD12				0.976	>0.9999	103	0.85	0.8869	47	0.628	0.023	37	0.819	0.176
50 μM AD14	0.589	0.0237	39	0.936	0.9985	106	0.58	0.006	68	0.644	0.0264	41	0.688	0.168
25 μM AD16	0.876	0.899	32	0.679	0.0261	108	0.59	0.0076	67	0.505	0.0001	54	0.662	0.159
50 μM AD29				0.646	0.0133	98	0.48	0.0002	68	0.544	0.0005	56	0.556	0.085
25 μM AD32				1.003	>0.9999	83	0.61	0.014	66	0.564	0.0202	23	0.726	0.241
