# Supplementary material for: Identification and characterization of small molecule inhibitors of the LINE-1 retrotransposon endonuclease
Source: Nat Commun. 2024 May 8;15:3883. doi: 10.1038/s41467-024-48066-x (PMC11078990; doi:10.1038/s41467-024-48066-x)
Supplement: Supplementary file 3 — Description of Additional Supplementary Files [file 41467_2024_48066_MOESM3_ESM.pdf]

Description of Additional Supplementary Files

File Name: Supplementary Data 1
Description: Oligonucleotide sequences. The oligonucleotides used in this study are listed showing their exact sequences. Also included for each oligonucleotide is the experiment/assay in which it was used.
