# Supplementary material for: Identification and characterization of small molecule inhibitors of the LINE-1 retrotransposon endonuclease
Source: Nat Commun. 2024 May 8;15:3883. doi: 10.1038/s41467-024-48066-x (PMC11078990; doi:10.1038/s41467-024-48066-x)
Supplement: Supplementary file 5 — Reporting Summary [file 41467_2024_48066_MOESM5_ESM.pdf]

Corresponding author(s): John M. Sedivy, Gerwald Jogl

Last updated by author(s): Mar 22, 2024

Reporting Summary

Nature Portfolio wishes to improve the reproducibility of the work that we publish. This form provides structure for consistency and transparency in reporting. For further information on Nature Portfolio policies, see our Editorial Policies and the Editorial Policy Checklist.

Statistics

For all statistical analyses, confirm that the following items are present in the figure legend, table legend, main text, or Methods section.

n/a	Confirmed	
☐	☒	The exact sample size (*n*) for each experimental group/condition, given as a discrete number and unit of measurement
☐	☒	A statement on whether measurements were taken from distinct samples or whether the same sample was measured repeatedly
☐	☒	The statistical test(s) used AND whether they are one- or two-sided<br>*Only common tests should be described solely by name; describe more complex techniques in the Methods section.*
☒	☐	A description of all covariates tested
☐	☒	A description of any assumptions or corrections, such as tests of normality and adjustment for multiple comparisons
☐	☒	A full description of the statistical parameters including central tendency (e.g. means) or other basic estimates (e.g. regression coefficient) AND variation (e.g. standard deviation) or associated estimates of uncertainty (e.g. confidence intervals)
☐	☒	For null hypothesis testing, the test statistic (e.g. *F*, *t*, *r*) with confidence intervals, effect sizes, degrees of freedom and *P* value noted<br>*Give P values as exact values whenever suitable.*
☒	☐	For Bayesian analysis, information on the choice of priors and Markov chain Monte Carlo settings
☒	☐	For hierarchical and complex designs, identification of the appropriate level for tests and full reporting of outcomes
☒	☐	Estimates of effect sizes (e.g. Cohen's *d*, Pearson's *r*), indicating how they were calculated

Our web collection on statistics for biologists contains articles on many of the points above.

Software and code

Policy information about availability of computer code

Data collection	Retrotransposition assay and fluorescent oligonucleotide assay: BioTek Gen5 software for Cytation 5 and Synergy H1 plate readers, versions 3.08 to 3.12.<br>Spectral shift: NanoTemper MO.Control software version v2.5.4 for Monolith X.<br>Fluorescence microscopy: Nikon NIS-Elements software 5.20.01 for Nikon Ti2-E Fluorescence Microscope.<br>X-ray crystallography: Brookhaven National Laboratory National Synchrotron Light Source II Automated Macromolecular Crystallography beamline Eiger 9M detector and Life Science Data Collection software.<br>RT-qPCR: Applied Biosystems ViiA 7 Real-Time PCR System or QuantStudio version 6 Pro.
Data analysis	General calculations (qPCR Ct value transformations, fluorescent oligonucleotide initial rates, average IC50 values, etc): Microsoft Excel 365<br>All statistical tests, IC50 and Kd curve fits, and graphs/plots/data visualizations: GraphPad Prism for Windows versions 9.4.1 to 10.2.0.<br>Molecular docking: LeDock version 1.0, AutoDock Vina version 1.1.2, DOCK version 6.9, FitDock version v1.0.1.<br>X-ray crystallography: XDS, Aimless in CCP4 version 8.0, Phaser and phenix.refine in Phenix version 1.20.1-4487, Coot version 0.9.8.8, PyMOL version 2.5.2.<br>Spectral shift: NanoTemper MO.Control software version v2.5.4 for Monolith X.<br>Microscopy image viewing and figure generation: Nikon NIS-Elements Viewer software 5.21, Adobe Photoshop 23.3.1<br>Fluorescence microscopy image quantification: Cell Profiler version 4.2.1.<br>Neutral comet assay quantification: OpenComet plugin version 1.3.1 for ImageJ version 1.53k.<br>RNA-seq analysis: fastp version v0.23.4, STAR version 2.7.3a, featureCounts in Subread version 2.0.3, DESeq2 in R version 4.3.0, and GSEA version 4.3.2.

For manuscripts utilizing custom algorithms or software that are central to the research but not yet described in published literature, software must be made available to editors and reviewers. We strongly encourage code deposition in a community repository (e.g. GitHub). See the Nature Portfolio guidelines for submitting code & software for further information.

Data

Policy information about availability of data

All manuscripts must include a data availability statement. This statement should provide the following information, where applicable:

- Accession codes, unique identifiers, or web links for publicly available datasets
- A description of any restrictions on data availability
- For clinical datasets or third party data, please ensure that the statement adheres to our policy

The x-ray crystallography data generated and protein structures solved in this study have been deposited in the Protein Data Bank under the following accession codes: 8SP5 [https://doi.org/10.2210/pdb8SP5/pdb] (LINE-1 retrotransposon endonuclease domain complex with Mn2+) and 8SP7 [https://doi.org/10.2210/pdb8SP7/pdb] (LINE-1 retrotransposon endonuclease domain complex with tranexamic acid,). The RNA-seq data generated in this study have been deposited in the Gene Expression Omnibus (GEO) database under accession code GSE244265 [https://www.ncbi.nlm.nih.gov/geo/query/acc.cgi?acc=GSE244265]. All other data are included in the Supplementary Information, Supplementary Data, or Source Data files provided with this paper.

Research involving human participants, their data, or biological material

Policy information about studies with human participants or human data. See also policy information about sex, gender (identity/presentation), and sexual orientation and race, ethnicity and racism.

Reporting on sex and gender	This research did not involve human participants, their data, or their biological material.
Reporting on race, ethnicity, or other socially relevant groupings	This research did not involve human participants, their data, or their biological material.
Population characteristics	This research did not involve human participants, their data, or their biological material.
Recruitment	This research did not involve human participants, their data, or their biological material.
Ethics oversight	This research did not involve human participants, their data, or their biological material.

Note that full information on the approval of the study protocol must also be provided in the manuscript.

Field-specific reporting

Please select the one below that is the best fit for your research. If you are not sure, read the appropriate sections before making your selection.

☒ Life sciences ☐ Behavioural & social sciences ☐ Ecological, evolutionary & environmental sciences

For a reference copy of the document with all sections, see nature.com/documents/nr-reporting-summary-flat.pdf

Life sciences study design

All studies must disclose on these points even when the disclosure is negative.

Sample size	Sample sizes are included in the figure legends for all experiments and were based on previously published results from similar experiments. Retrotransposition assay measurements were completed in quadruplicate based on previous uses of the retrotransposition assay with the pPM404 plasmid (Mita et al. 2020). DNA damage assays were completed with sample sizes greater than 15 nuclei per treatment to account for the biological variability of individual cells. Senescent cell assays were completed in triplicate as previously done in the Sedivy laboratory (DeCecco et al. 2019). All other measurements were performed in triplicate as is standard.
Data exclusions	For the y-H2AX immunofluorescence quantification, the ROUT outlier correction with Q=0.1% was applied to remove extreme outliers from staining artifacts in an unbiased manner. This criteria was established during assay development and optimization prior to treatment with EN inhibitors.
Replication	The data shown represents results that were reproducible across multiple independent experiments. Figure and table legends indicate whether graphs display representative results from one independent experiment or multiple independent experiments. All results were reproducible.
Randomization	Individual wells or plates of cells were treated identically prior to the experiment and then were allocated to each experimental group randomly.
Blinding	Image fields for microscopy were chosen randomly. Microscopy image quantification was completed automatically by Cell Profiler or OpenComet, so blinding was not relevant at this stage. For all other experiments blinding was not relevant as measurements and calculations were objectively completed by instrumentation and/or software.

Reporting for specific materials, systems and methods

We require information from authors about some types of materials, experimental systems and methods used in many studies. Here, indicate whether each material, system or method listed is relevant to your study. If you are not sure if a list item applies to your research, read the appropriate section before selecting a response.

Materials & experimental systems

n/a	Involved in the study	
☐	☒	Antibodies
☐	☒	Eukaryotic cell lines
☒	☐	Palaeontology and archaeology
☒	☐	Animals and other organisms
☒	☐	Clinical data
☒	☐	Dual use research of concern
☒	☐	Plants

Methods

n/a	Involved in the study	
☒	☐	ChIP-seq
☒	☐	Flow cytometry
☒	☐	MRI-based neuroimaging

Antibodies

Antibodies used

All antibodies described in this work were used for immunofluorescence microscopy.

Human LINE-1 ORF1 rabbit primary antibody: Abcam, rabbit monoclonal, cat. no. ab245249, clone EPR22227-6.
Human LINE-1 ORF1 mouse primary antibody: Millipore, mouse monoclonal, cat. no. MABC1152, clone 4H1.
y-H2AX primary antibody: Millipore, mouse monoclonal, cat. no. 05-636, clone JBW301.
DNA/RNA hybrids primary antibody: Kerafast, mouse monoclonal, cat. no. ENH001, clone S9.6.
Alexa Fluor 488 secondary antibody: Thermo Fisher, goat anti-rabbit polyclonal, cat. no A-11008
Alexa Fluor 546 secondary antibody: Thermo Fisher, donkey anti-mouse polyclonal, cat. no A10036
Alexa Fluor 647 secondary antibody: Thermo Fisher, donkey anti-mouse polyclonal, cat. no A-31571

Validation

All antibodies are commercially available and have been validated for use in immunofluorescence experiments by the vendor as shown by supporting documentation and publications referenced on vendor websites. See below for additional details. We also confirmed antibody activity and optimized dilutions in our laboratory using appropriate positive and negative controls.

Human LINE-1 ORF1 rabbit
Species reactivity: Human
Validated applications: Western Blotting, Immunohistochemistry, Immunocytochemistry/Immunofluorescence, Immunoprecipitation, Flow Cytometry (Intracellular)

Human LINE-1 ORF1 mouse
Species reactivity: Human
Validated applications: Western Blotting, Immunocytochemistry/Immunofluorescence, Immunoprecipitation, Immunohistochemistry

y-H2AX
Species reactivity: Vertebrates
Validated applications: Immunocytochemistry/Immunofluorescence, Western Blotting, Chromatin Immunoprecipitation, Immunohistochemistry

DNA/RNA hybrids
Species reactivity: Species Independent
Validated applications: Dot Blot, Affinity Binding Assay, ChIP, Immunocytochemistry/Immunofluorescence, Immunohistochemistry, Immunoprecipitation

Eukaryotic cell lines

Policy information about cell lines and Sex and Gender in Research

Cell line source(s)

LF1 cells used for the senescence experiments were originally harvested from embryonic lung tissue (Brown et al., Science, 1997) and have been used in the Sedivy laboratory since then. HeLa Tet-On cells were purchased from Takara Bio Inc. and HeLa Tet-On cells containing pPM404 were a gift from the laboratory of Jef Boeke.

Authentication

LF1 fibroblasts were authenticated as having no contamination by ATCC STR Profiling Service in 2019. HeLa Tet-On cells were tested by Takara Bio using functional assays as described in the Certificate of Analysis and used at low passage in our laboratory. HeLa Tet-On cells containing pPM404 were not further authenticated upon receipt from the laboratory of Jef Boeke and were used at low passage in our laboratory.

Mycoplasma contamination

HeLa Tet-On cells were confirmed to be free from mycoplasma contamination by Takara Bio Inc. according to the Certificate of Analysis provided on their website. In our laboratory all cultures were tested regularly for mycoplasma contamination and

	tested negative.
Commonly misidentified lines<br>(See ICLAC register)	None used.
